# Probiotic Lactobacilli Do Not Protect Chickens against *Salmonella* Enteritidis Infection by Competitive Exclusion in the Intestinal Tract but in Feed, Outside the Chicken Host

**DOI:** 10.3390/microorganisms10020219

**Published:** 2022-01-20

**Authors:** Helena Juricova, Jitka Matiasovicova, Marcela Faldynova, Alena Sebkova, Tereza Kubasova, Hana Prikrylova, Daniela Karasova, Magdalena Crhanova, Hana Havlickova, Ivan Rychlik

**Affiliations:** Veterinary Research Institute, 621 00 Brno, Czech Republic; juricova@vri.cz (H.J.); matiasovicova@vri.cz (J.M.); faldynova@vri.cz (M.F.); asebkova@vri.cz (A.S.); kubasova@vri.cz (T.K.); prikrylova@vri.cz (H.P.); karasova@vri.cz (D.K.); crhanova@vri.cz (M.C.); havlickova@vri.cz (H.H.)

**Keywords:** *Lactobacillus*, *Salmonella* Enteritidis, chicken, probiotic, feed fermentation, gut microbiota

## Abstract

Lactobacilli are commonly used as probiotics in poultry to improve production parameters and to increase chicken resistance to enteric infections. However, lactobacilli do not efficiently colonise the chicken intestinal tract, and also, their anti-infection effect in vivo is sometimes questionable. In this study, we therefore evaluated the potential of a mixture of four *Lactobacillus* species (*L. salivarius*, *L. reuteri*, *L. ingluviei* and *L. alvi*) for the protection of chickens against *Salmonella* Enteritidis infection. Whenever the chickens were inoculated by lactobacilli and *S*. Enteritidis separately, there was no protective effect of lactobacilli. This means that when lactobacilli and *S*. Enteritidis are exposed to each other as late as in the crop of chickens, lactobacilli did not influence chicken resistance to *S.* Enteritidis at all. The only positive effect was recorded when the mixture of lactobacilli and *S*. Enteritidis was used for the inoculation of feed and the feed was anaerobically fermented for 1 to 5 days. In this case, chickens fed such a diet remained *S*. Enteritidis negative. In vitro experiments showed that the protective effect was caused by acidification of feed down to pH 4.6 due to lactobacilli fermentation and was associated with *S*. Enteritidis inactivation. The probiotic effect of lactobacilli was thus expressed in the feed, outside the chicken host.

## 1. Introduction

Gut microbiota is at present a subject of high interest [1,2]. This interest is caused by the well-recognised role of gut microbiota for their hosts and, simultaneously, by technological developments in nucleic acid sequencing. Massive parallel sequencing now allows the determination of the structure of any microbial population, including that from the intestinal tract. The gut microbiota of warm-blooded animals consists of hundreds of bacterial species. Of those well-known to humans, some might be associated with disorders and disbalances, such as *Helicobacter*, *Campylobacter* or *Clostridium perfringens* [3], whilst others such as *Lactobacillus*, *Bifidobacterium* and other lactic acid bacteria are considered beneficial microbiota members [4].

The term lactic acid bacteria refers to a heterogeneous group of bacteria that produce lactic acid as the main product of their carbohydrate fermentation. Lactobacilli are facultative anaerobes or aerotolerant bacteria commonly present in the external environment [5,6]. Due to this fact, lactobacilli have been consumed by humans for centuries through food and are therefore regarded as safe [7]. Lactobacilli withstand the acidic environment of the stomach and grow in the harsh but nutrient-rich environment of the proximal gastrointestinal tract [8]. In the duodenum, jejunum and ileum, lactobacilli may form up to 90% of all microbiota [9,10] but their abundance decreases in distal parts of the intestinal tract and, consequently, lactobacilli form only around 1% of total microbiota in the colon, caecum and faeces [8,11]. Lactobacilli also colonise additional compartments of humans and other animals such as the oral cavity, respiratory tract or vagina [12].

Lactobacilli are characterised by rather small genomes of around 2 Mbp in size and low genomic GC content [13]. Lactobacilli encode various cell-surface proteins and structural components implicated in adherence to mucus and epithelial cells and in signalling to immune and dendritic cells of the intestinal mucosa [14]. Lactobacilli may therefore adhere to enterocytes and actively modulate the host immune response. Moreover, lactobacilli produce lactic acid and other metabolites (short-chain organic acids, bacteriocins, hydrogen peroxide), which can suppress the growth of pathogens within the intestinal tract. However, while the antimicrobial activity of lactobacilli against various foodborne bacterial pathogens, such as *Campylobacter jejuni*, *Listeria monocytogenes*, *Escherichia coli* and *Salmonella enterica* is well established in vitro [15,16,17,18,19], simple identification of bacteriocins or other growth-suppressing metabolites in vitro does not guarantee an in vivo effect. The probiotic effect of lactobacilli is therefore declared as strain-dependent and probiotic therapy usually requires daily administration of high doses (10^9^–10^10^ per dose) of lactobacilli [12,20]. Despite this, there are studies summarising that lactobacilli-derived probiotics are sometimes of questionable efficacy [4].

In the poultry industry, commercial probiotic products containing lactobacilli are used to improve production parameters including egg weight, body weight or feed conversion ratio [21,22]. Lactobacilli are used also to prevent enteric diseases [18]. However, when we followed the fate of individual strains of lactobacilli after a single oral inoculation of chicks on the day of hatching, these were not detected in the caecum of inoculated chicks one week later [23]. Considering the number of reports on the positive effect of lactobacilli for gut health, it was rather unexpected that seven of seven tested strains originally isolated from poultry did not colonise a naive environment such as the caecum of chicks during the first week of life. Consequently, lactobacilli-administered chicks were not protected against *Salmonella enterica* serovar Enteritidis (*S.* Enteritidis) challenge [23].

The aim of this study therefore was to clarify the probiotic effect of lactobacilli in chickens. As a model, the chicken–lactobacilli–*Salmonella* interaction was selected. Specifically, we tested whether lactobacilli at least partially protected chicks against *S.* Enteritidis infection. We tested orally administered lactobacilli in liquid suspension or via fermented feed followed by *S.* Enteritidis challenge one week later. We also tested the consequences of parallel administration of lactobacilli and *S.* Enteritidis. Furthermore, in vivo data was finally confirmed by co-culture experiments in vitro. Our data showed that if *S.* Enteritidis is provided in parallel to lactobacilli culture, there was no protection. The only protective effect of lactobacilli was recorded in experiments in which the feed was inoculated both by lactobacilli and *Salmonella* and fermented for at least 24 h before providing to chickens.

## 2. Materials and Methods

### 2.1. Bacterial Strains

Four different *Lactobacillus* isolates, *L. alvi* An810, *L. ingluviei* An777, *L. reuteri* An769, and *L. salivarius* An63, obtained from chicken caeca were used in this study. The strains were characterised by whole genomic sequencing previously [13] and their genomic sequences are deposited in NCBI under Bioproject accession number PRJNA377666. Their genomic sequences were interrogated for bacteriocin-encoding genes using BAGEL4 software available at http://bagel4.molgenrug.nl/index.php. The taxonomic classification of lactobacilli is under development [24] and *L. alvi*, *L. ingluviei* and *L. reuteri* have been reclassified into genus *Limosilactobacillus* with retained species names. *L. salivarius* has been renamed to *Ligilactobacillus salivarius*. However, for the purpose of this study, the original taxonomy is used for clarity. *S.* Enteritidis 147, spontaneously resistant to nalidixic acid, is originally a chicken isolate of phage type PT4 [25]. The *E. coli* ET76 strain used in in vitro experiments was obtained from chickens.

### 2.2. Experimental Animals

In all experiments, newly hatched male ISA Brown chicks were obtained from a local hatchery on the day of hatching. Chicks were reared in plastic boxes with free access to water and feed in rooms with a controlled light and temperature regime and filtered air supply. Rearing conditions corresponded to those generally recommended for rearing chicks during the first days of life.

### 2.3. Lactobacilli Administration and S. Enteritidis Challenge

Three independent experiments with different routes of lactobacilli and *S.* Enteritidis administration were performed. In the first experiment, oral administration of liquid lactobacilli cultures was tested. Four lactobacilli isolates were grown separately in 5 mL of Brain Heart Infusion (BHI) at 37 °C in a Bactron600 anaerobic cabinet (Sheldon Manufacturing Inc., Cornelius, OR, USA). After 24 h cultivation, equal volumes of all 4 cultures were mixed, pelleted by centrifugation and resuspended in phosphate-buffered saline. The suspension was mixed with drinking water so that the final concentration of each *Lactobacillus* species in drinking water was 10^7^ CFU/mL. Drinking water with lactobacilli was provided to chicks (*n* = 15) from day 1 to day 8 of life by replacing fresh water with fresh lactobacilli cultures on a daily basis. Chicks in the control group (*n* = 10) were kept in a separate room without any lactobacilli treatment. On day 8, five chicks from both the lactobacilli-treated and control group were euthanised to check for lactobacilli colonisation of the caecum, and the remaining chicks were orally challenged with 10^7^ CFU of *S.* Enteritidis in 0.1 mL inoculum. After challenge, the chicks from the lactobacilli-treated group were divided into two subgroups with 5 chicks in each group. In the first group, the administration of lactobacilli was discontinued and in the second group, daily administration of lactobacilli via drinking water continued until the end of the experiment. The experiment was terminated 4 days post *S.* Enteritidis infection.

In the second experiment, oral administration of lactobacilli via fermented feed was tested. To reach this aim, 15 g of feed was sterilised by autoclaving for 20 min at 120 °C. In parallel, lactobacilli cultures were re-suspended in 15 mL of sterile BHI to a final concentration of 10^7^ CFU/mL for each of the strains, and the whole volume was immediately used to moisten the feed to inoculate it with lactobacilli. The feed was then incubated anaerobically for 24 h at 37 °C, after which the fermented feed was provided to newly hatched chicks (*n* = 10) for the first 3 days of life. A fresh batch of the fermented feed was provided to the chicks daily. From day 4 of life, chicks in the experimental group were provided a standard dry granulated diet. Chicks in the control group (*n* = 10) were provided a standard dry granulated diet throughout the whole experiment, without any lactobacilli supplementation. Similar to the first experiment, five chicks from both groups were euthanised to check for lactobacilli colonisation on day 8, and the remaining chicks were challenged with *S.* Enteritidis, as described above. The experiment was terminated 4 days post infection with *S.* Enteritidis when the chicks were 12-days old.

In the last experiment, the effect of co-fermentation of lactobacilli and *S.* Enteritidis was tested. Fifteen grams of feed was autoclaved and moistened with 15 mL of sterile BHI inoculated with 4 lactobacilli isolates, each at 10^7^ CFU/mL concentration, as well as with 10^5^ CFU/mL *S.* Enteritidis. The feed was then anaerobically incubated for either 1 or 5 days. Just before administration to chickens, 0.5 g of the fermented feed was taken to determine pH and enumerate *S.* Enteritidis. The feed, fermented either for 1 or 5 days, was provided to two groups of chickens, each consisting of 7 newly hatched chicks, for the first 3 days of life. Chicks in the control group (*n* = 7) were given only lactobacilli-fermented feed (incubated for 24 h) and *S.* Enteritidis was supplied in drinking water at a concentration of 10^5^ CFU/mL for the first 3 days of life. From day 4, chicks in all groups were provided a standard dry granulated diet and drinking water free of lactobacilli and *S.* Enteritidis. Chicks in all groups were sacrificed on day 8 of life.

### 2.4. Processing of Chicken Caecal and Liver Samples for S. Enteritidis Enumeration

After termination of each of the experiments, 0.5 g caecal content and liver tissue were removed, homogenised in 5 mL peptone water, serially diluted and plated on xylose lysine deoxycholate (XLD) agar supplemented with nalidixic acid. *S.* Enteritidis colonies were counted after 48 h of aerobic incubation at 37 °C. In the case of no *Salmonella* colonies after direct plating, peptone water homogenates were processed according to ISO 6579 protocol for qualitative *Salmonella* detection. *S.* Enteritidis counts were logarithmically transformed and samples positive only after the ISO protocol were assigned a value of 1 and negative samples were given a value of 0.

### 2.5. Real-Time PCR Detection of S. Enteritidis and Each Lactobacillus Isolate

The contents of paired caeca were collected and frozen at −20 °C for DNA extraction and *Salmonella* and *Lactobacillus* quantification by real-time PCR. DNA from caecal samples was extracted using a QIAamp Stool kit according to the manufacturer’s instructions (Qiagen, Hilden, Germany). Based on known genomic sequences, strain-specific real-time PCRs were designed to determine colonisation of the caecum by *S.* Enteritidis and each of the lactobacilli isolates (Table 1). Real-time PCR in SybrGreen format was performed exactly as described previously [26].

### 2.6. In Vitro Lactobacilli and Salmonella Co-Cultivation in Feed

Fifteen grams of feed were sterilised by autoclaving at 120 °C for 30 min. The sterile feed (15 g) was moistened with 15 mL of BHI, which was inoculated with an overnight culture of *S.* Enteritidis (10^8^ CFU/mL) and/or lactobacilli (10^8^ CFU/mL) prior to mixing with the feed. The ratio of *S.* Enteritidis and lactobacilli in the inocula is shown for each experiment below. The feed was anaerobically incubated at 37 °C for 1 to 4 days as specified in the text. Following incubation, bacterial counts were determined by serial dilution and plating on XLD and Wilkins–Chalgren agar plates (WCHA). XLD plates were incubated aerobically, thus allowing only for *S.* Enteritidis growth since control experiments showed no growth of any of 4 lactobacilli strains on XLD under aerobic conditions (not shown). Anaerobic incubation of WCHA allowed for growth of both lactobacilli and *Salmonella* but knowing *Salmonella* counts from XLD plates, it was possible to determine the lactobacilli count by subtracting XLD *Salmonella* counts from total bacterial counts on WCHA agar plates. Since the XLD and WCHA counts differed in logs of magnitude, the definition of lactobacilli and *Salmonella* counts was quite clear.

### 2.7. Statistics

A *t*-test or ANOVA followed by post hoc Tuckey’s test were used to evaluate *S.* Enteritidis counts in the caecum and liver of control and experimental chickens. Comparisons with *p* < 0.05 were considered as significantly different.

### 2.8. Ethics Approval

The handling of animals in the study was performed in accordance with current Czech legislation (Animal Protection and Welfare Act No. 246/1992 Coll. of the Government of the Czech Republic). The specific experiments were approved by the Ethics Committee of the Veterinary Research Institute followed by the Committee for Animal Welfare of the Ministry of Agriculture of the Czech Republic (permit number MZe1922 approved on 15 January 2018).

## 3. Results

### 3.1. Oral Administration of Lactobacilli in Liquid Cultures

**Figure 1:**
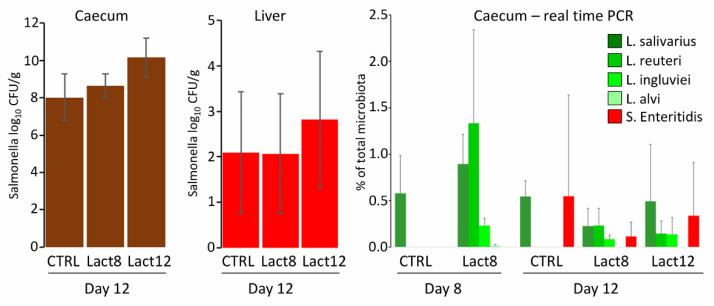
Oral administration of lactobacilli and their effect on the resistance of chickens to *S.* Enteritidis. *S.* Enteritidis counts in the liver and caecum did not differ in control (CTRL) or experimental chickens given lactobacilli for 8 days only prior to *S.* Enteritidis infection (Lact8) or throughout the whole experiment (Lact12). The presence of lactobacilli and *S.* Enteritidis used in this experiment was determined by real-time PCR performed both on day 8 and day 12 of life, showing a similar abundance of lactobacilli and *S.* Enteritidis in the tested caeca.

In the first experiment, the effect of daily oral administration of liquid lactobacilli cultures on chicken resistance to *S.* Enteritidis was tested. Of the four used lactobacilli species, three of them were detected in the caecum by species-specific PCR. *L. alvi*, despite daily administration, was not detected in the caecum at all. *L. salivarius*, though recorded in the caeca of experimental chickens, was present also in the caeca of control chickens, so it was impossible to determine whether its presence in the experimental chickens originated from experimental administration or whether the chicks in the experimental group were colonised by *L. salivarius* of environmental origin, as happened in the control chicks. Regardless of lactobacilli colonisation status in the caecum, high *S.* Enteritidis counts were recorded in the caecum and liver of control or experimental chicks and these did not differ significantly among the groups, even in the case when lactobacilli were provided daily throughout the whole 12 days of the experiment (Figure 1).

Since absolute *S.* Enteritidis counts were difficult to relate to lactobacilli abundance determined by real-time PCR, *S.* Enteritidis abundance was determined by real-time PCR as well. This showed that *S.* Enteritidis abundance in the total microbiota was similar to that of lactobacilli and the absence of the probiotic effect of lactobacilli cannot be explained by dominance of *S.* Enteritidis over lactobacilli or insufficient lactobacilli abundance in the chicken caecum.

### 3.2. Oral Administration of Lactobacilli via Fermented Feed

**Figure 2:**
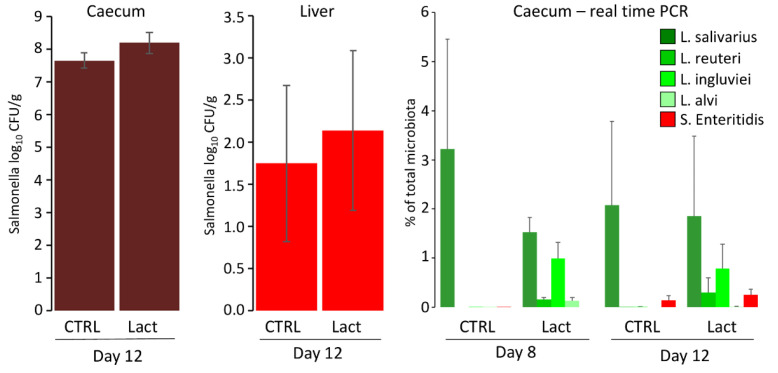
Oral administration of lactobacilli via fermented feed and its effect on chicken resistance to *S.* Enteritidis infection. *S.* Enteritidis counts in the liver and caecum did not differ in control or experimental chickens. The presence of lactobacilli and *S.* Enteritidis used in the experiment was determined by real-time PCR performed both on day 8 prior to *S.* Enteritidis infection and on day 12 when the experiment was terminated.

The protective effect of orally administered lactobacilli via fermented feed was tested in a second experiment. Similar to the previous experiment, *L. alvi* colonised the chicken caecum the least. It was impossible to determine whether colonisation of *L. salivarius* was a consequence of administration or its presence in the environment (Figure 2). Similar results were also recorded for S. Enteritidis, since *S.* Enteritidis counts in the caecum and liver did not differ significantly between experimental and control chickens (Figure 2). When *S.* Enteritidis abundance was determined by real-time PCR, *S.* Enteritidis abundance was lower than the abundance of lactobacilli. The absence of a probiotic effect could not be explained by insufficient lactobacilli counts in the chicken caecum.

### 3.3. Oral Administration of Lactobacilli and S. Enteritidis via Co-Fermented Feed

**Figure 3:**
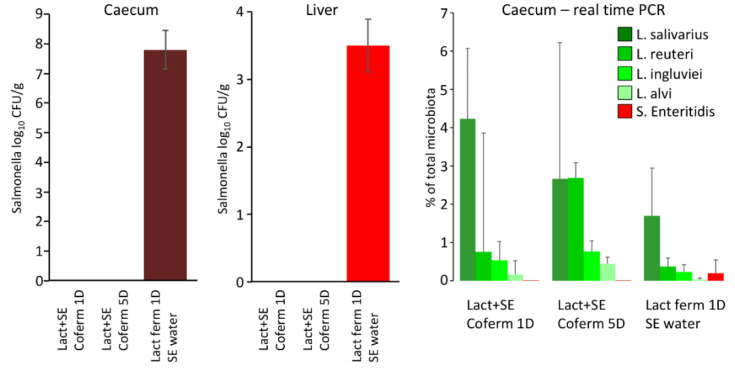
Effect of co-fermentation of lactobacilli and *S.* Enteritidis for chicken colonisation with *S.* Enteritidis. Chicks provided lactobacilli-fermented feed but with *S.* Enteritidis administered via drinking water, were highly positive for *S.* Enteritidis in the liver and caecum. However, those provided fermented feed inoculated with both *S.* Enteritidis and lactobacilli were negative for *S.* Enteritidis. All treated chicks were positive for the used lactobacilli and also for *S.* Enteritidis in the chickens inoculated with *S.* Enteritidis via drinking water as determined by real-time PCR.

The effect of co-fermentation of lactobacilli and *S*. Enteritidis was tested in the last in vivo experiment. As in both previous experiments, *L. alvi* colonised chicken caecum the least and *L. salivarius* colonised it the most. When *S.* Enteritidis counts were determined in the chicken caeca, only chickens provided lactobacilli-fermented feed and *S.* Enteritidis via drinking water were *S.* Enteritidis positive. This was also confirmed when *S.* Enteritidis abundance was determined by real-time PCR (Figure 3).

On the other hand, chickens provided the feed inoculated by both *S.* Enteritidis and lactobacilli, irrespective of whether it was co-fermented for 1 or 5 days, were *S.* Enteritidis negative (Figure 3). However, the control culture showed that feed co-fermented by both lactobacilli and *S.* Enteritidis for 1 or 5 days was negative for *S.* Enteritidis (not shown). This, of course, explains the subsequent negativity of chickens for *S.* Enteritidis.

### 3.4. Co-Culture of S. Enteritidis and Lactobacilli In Vitro

**Figure 4:**
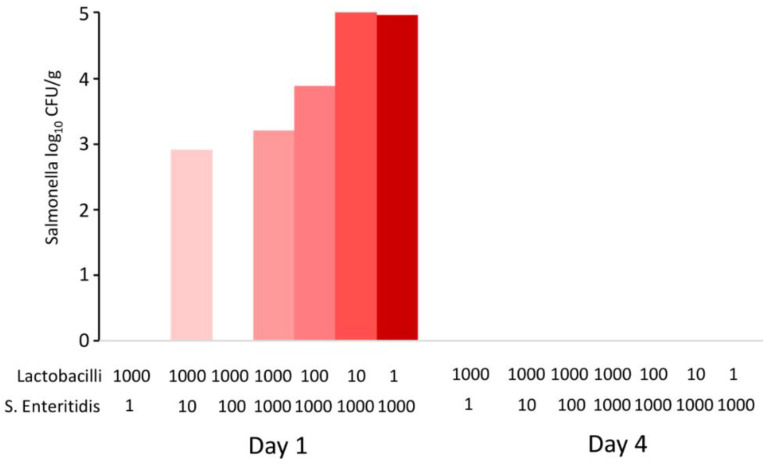
In vitro co-culture of *S.* Enteritidis and lactobacilli. Sterile feed (15 g) was moistened with 15 mL of BHI with different initial ratios of *S.* Enteritidis and lactobacilli (defined by volumes in microliters of inocula of stock cultures with 10^8^ CFU/mL). *S.* Enteritidis was completely inactivated in the feed by day 4, even in the samples in which it initially dominated over lactobacilli.

Data from in vivo experiments indicated that the probiotic effect of lactobacilli was expressed outside the host, during feed fermentation. Since the last in vivo experiment also lacked some controls, such as controlling for the growth of *S.* Enteritidis in the feed alone, multiple in vitro experiments were subsequently performed. In the first experiment, the feed was inoculated with different initial ratios of lactobacilli and *S.* Enteritidis and incubated for 1 and 4 days. After 24 h culture, the pH dropped to values around pH 4.8 and decreased further to pH 4.6 by day 4. When *S.* Enteritidis dominated over lactobacilli in the inoculum, *S.* Enteritidis was detected in the feed after 24 h of co-culture. However, even in the feed samples inoculated with *S.* Enteritidis dominating by three logs over lactobacilli, *S.* Enteritidis disappeared completely by day 4 of co-culture (Figure 4).

**Figure 5:**
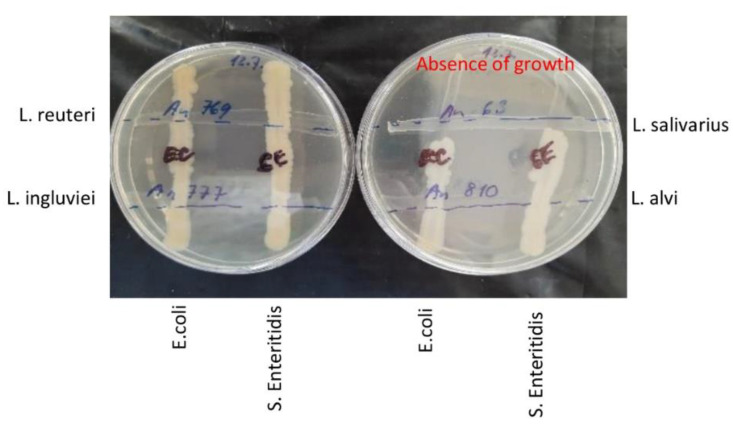
Inhibitory effect of 4 *Lactobacillus* species against *S.* Enteritidis and *E. coli* on agar plates. Lactobacilli strains were streaked over the WCHA agar plates and incubated anaerobically for 24 h. Then, *S.* Enteritidis and *E. coli* were streaked across lactobacilli and the incubation was extended for an additional 48 h. *L. salivarius* was the only *Lactobacillus* isolate which inhibited the growth of both *S.* Enteritidis and *E. coli*.

In an alternate experiment, all four lactobacilli were grown for 24 h on WCHA agar plates, and *S.* Enteritidis and *E. coli* cultures were then streaked across already grown lactobacilli. Following an additional incubation for 48 h, *L. salivarius* suppressed *S.* Enteritidis and *E. coli* growth, while the remaining three *Lactobacillus* strains did not (Figure 5). As we determined the whole genomic sequences of all four *Lactobacillus* strains, a search for the presence of genes encoding antimicrobial peptides was performed. We found that *L. salivarius* harboured the gene encoding bacteriocin LS2 [27]. Therefore, a series of experiments to determine the role of pH or other metabolites with potential antimicrobial properties in the suppression of *S.* Enteritidis multiplication was performed.

**Figure 6:**
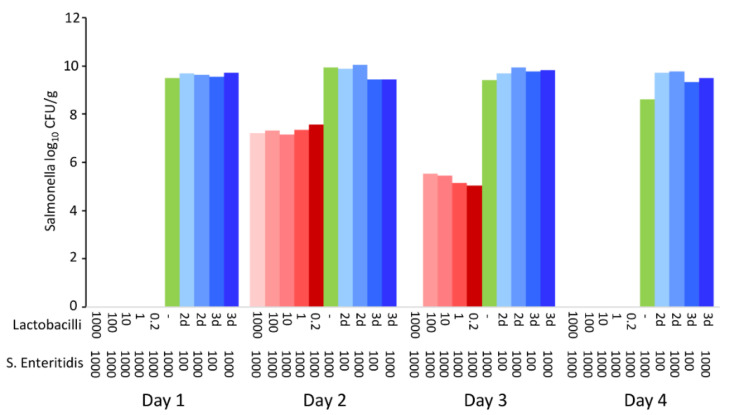
Comparison of anti-*S*. Enteritidis effects of viable lactobacilli or lactobacilli cell-free supernatant. While viable lactobacilli efficiently inactivated *S.* Enteritidis during co-culture irrespective of the initial ratio of lactobacilli to *S.* Enteritidis shown in µL (shades of red), cell-free supernatants from 2- or 3-day-old lactobacilli culture were ineffective against *S.* Enteritidis, irrespective of whether 100 or 1000 µL of *S.* Enteritidis was used for inoculation (shades of blue). If only *S.* Enteritidis was used for moistened-feed inoculation, it grew on this substrate up to 10^10^ CFU/g of feed (green column).

First, the activity of cell-free supernatants against *S.* Enteritidis growth was tested. Supernatants were collected from 2- and 3-day-old cultures of all lactobacilli, the filter sterilised and the pH adjusted to 6.7 by NaOH to eliminate any pH effect. The supernatants did not affect *S.* Enteritidis growth, while viable lactobacilli caused the pH to decrease and *S.* Enteritidis was inactivated within 4 days of incubation (Figure 6).

**Figure 7:**
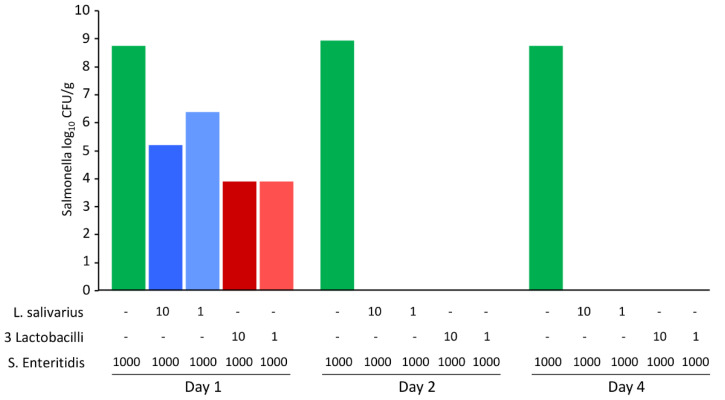
*S*. Enteritidis viability during co-culture with *L. salivarius* or a mixture of *L. alvi*, *L. ingluviei* and *L. reuteri*. Sterile feed (15 g) was mixed with 15 mL of BHI inoculated with different volumes (shown in µL) of *L. salivarius*, a mixture *L. alvi*, *L. ingluviei* and *L. reuteri* (3 lactobacilli) and *S.* Enteritidis. Although *L. salivarius* differed in its behaviour in the test using agar plates (Figure 5) and also in the presence of genes for antimicrobial peptides in its genome, these characteristics were dispensable for the suppression of *S.* Enteritidis multiplication in the fermented feed, which disappeared by day 2 and 4 in all tubes inoculated with both *S.* Enteritidis and any *Lactobacillus*.

Finally, *S.* Enteritidis inactivation in the feed separately by *L. salivarius* and the remaining three *Lactobacillus* species was tested. Two different initial ratios of lactobacilli to *S.* Enteritidis were tested, including the inoculation of the feed with *S.* Enteritidis only. In the absence of lactobacilli, *S.* Enteritidis multiplied in the moistened feed. However, when *S.* Enteritidis was seeded together with lactobacilli, regardless of whether it encoded genes for antimicrobial peptides or not, *S*. Enteritidis could not be detected in the feed after 2 days of co-culture (Figure 7).

## 4. Discussion

Lactobacilli are considered safe and beneficial bacteria for their hosts. Their beneficial effect is accepted generally, but under such circumstances, people tend to become less critical to recorded results. We have become rather critical towards lactobacilli as probiotics in poultry since we found out that different *Lactobacillus* species of chicken origin did not colonise the caecum of newly hatched chicks [23]. Although the probiotic effect can be expressed even without permanent colonisation, one would expect a higher likelihood of the positive effect associated with efficient gut colonisation.

It has been repeatedly confirmed that lactobacilli produce metabolites that suppress the growth of *Salmonella* [28,29,30]. Lactobacilli also produce metabolites affecting host gene expression [31,32]. We have also reported that whenever lactobacilli overgrow, they efficiently suppress growth of other gut microbiota members [15]. However, all these results were obtained in in vitro experiments and ignored the fact that *Lactobacillus* cannot encounter macrophages in the lumen of the ileum or caecum. Consequently, the probiotic effect of lactobacilli in chickens is less clear. There are reports on the positive effect of lactobacilli increasing chicken resistance to infection with different *Salmonella* serovars [33,34,35], as well as reports on the lack of effect of lactobacilli administration for increased resistance of chickens against *Salmonella* [36,37,38]. There are also studies which report a positive effect in one trial and no effect in the repeated trial [39,40]. Alternatively, some papers reported a significant difference in *Salmonella* counts between control and experimental chickens at one time point, the absence of significance in the next time point and the re-appearance of positive probiotic effect at the third sampling time point of the same experiment [41,42,43]. Finally, and as shown also in this study for *L. salivarius*, the environmental supply of lactobacilli is usually high enough to cover all the needs of chickens. The effect of additional lactobacilli supplementation may occur at levels already exceeding daily requirements. All of this indicates that the in vivo effect of lactobacilli against *Salmonella* in chickens is questionable, although it can be argued that each in vivo experiment was of different experimental design, such as the use of different *Lactobacillus* species, *Salmonella* serovars, and chicken lines (broilers or layers), as well as the age of chickens, and the timing and mode of administration of lactobacilli or *Salmonella*.

This is why we addressed the role of lactobacilli as probiotics in this study and showed that lactobacilli did not protect chickens against *S.* Enteritidis infection if lactobacilli and *S.* Enteritidis meet as late as in the crop of chickens, or beyond. On the other hand, lactobacilli were effective against *S.* Enteritidis if these two bacteria interacted in the feed, prior to ingestion by chickens. Such an observation may explain the conflicting results from other studies. If lactobacilli are continuously supplied via feed, which is a very common approach, lactobacilli can make the feed safer by suppressing the growth of additional microbiota in the feed, thus giving a minor advantage over the chickens fed a lactobacilli-free diet. Lactobacilli from the feed can be released in the litter, comprising around 5% of total microbiota [5,6,44,45], and may decrease *Salmonella* survival in the litter—thus reducing the probability of chicken faecal–oral reinfection. Since our experiments used a perforated floor with minimal litter retention in the chicken environment, this could have contributed to the absence of any effect of lactobacilli administration on chicken resistance to *S.* Enteritidis. Lactobacilli are also common members of the skin and respiratory tract microbiota [12,46], and lactobacilli continuously provided in the feed may also reach these compartments and provide chickens with a higher resistance to skin and respiratory tract infections. It is well known that immunocompromised individuals are more susceptible to secondary infections, including those of the intestinal tract. There is also a report showing that experimentally administered lactobacilli efficiently colonise the chicken crop [47]. If the viability of some *Salmonella* isolates can be suppressed by low pH in the crop, this may also explain some of the positive reports.

It cannot be excluded that there might be cases when specific lactobacilli isolates have a protective effect against infection with a particular *Salmonella* strain. In addition, the probiotic effect of lactobacilli cannot not be considered only according to resistance to *Salmonella* infection, since lactobacilli administration also affects body weight and feed conversion [48,49]. We are aware that only early resistance to *S.* Enteritidis infection 4 days post-infection was determined in this study, while chickens can be positive for two months after infection during the first week of life [50,51] and lactobacilli may shorten this period. Despite this, the probiotic effect of lactobacilli in vivo as an anti-*Salmonella* measure should be viewed more critically, while the anti-*Salmonella* effect of lactobacilli in food and feed fermentation was quite clear. 

## 5. Conclusions

When lactobacilli and *S*. Enteritidis are exposed to each other as late as in the crop of chickens, lactobacilli do not influence chicken resistance to *S.* Enteritidis. The only positive effect was recorded when lactobacilli and *S*. Enteritidis were exposed to each other in feed and the feed was anaerobically fermented for at least 1 day. The most likely explanation of the antimicrobial effect of lactobacilli in feed is their rapid multiplication associated with a decrease in pH. The production of additional metabolites with antimicrobial activity is of lower importance. Such a conclusion explains the observed effect in vitro and the absence of the effects in vivo, and can be equally valid for chickens as well as other farm animals, or even humans.

## Figures and Tables

**Table 1:** List of primers used for the quantification of *S.* Enteritidis and each of lactobacilli strains used in this study.

Strain	Forward Primer	Reverse Primer
*L. alvi* An810	AAGCAAACTGGCTGTCCATT	ACCAAGGTATCGCGACTGAT
*L. ingluviei* An777	AGTCCTCCACGAACATACCG	TGATTAGTGGCACCGTCAAA
*L. reuteri* An769	GAAGCAAAGCCAGCTCAAAC	TCCCCGGATTGTCAAAGTAG
*L. salivarius* An63	TCGATGACGTTTTCGGTGTA	AAAAGCCGTGTTCGACAATC
*Salmonella enterica*	CGTATTTTCTGGCGTAAGTC	TTAGGTCAAATAGGGCAGAC
Eubact. 16S rRNA	TCCTACGGGAGGCAGCAG	CGTATTACCGCGGCTGCT

## Data Availability

The authors declare that the data supporting the findings of this study are available within the article.
